# Doctors’ engagements with patient experience surveys in primary and secondary care: a qualitative study

**DOI:** 10.1111/hex.12465

**Published:** 2016-04-28

**Authors:** Conor Farrington, Jenni Burt, Olga Boiko, John Campbell, Martin Roland

**Affiliations:** ^1^ Cambridge Centre for Health Services Research (CCHSR)Institute of Public HealthForvie SiteUniversity of Cambridge School of Clinical MedicineCambridgeUK; ^2^ Department of Primary Care and Public HealthKing's College LondonLondonUK; ^3^ University of Exeter Medical SchoolExeterUK

**Keywords:** patient experience surveys, primary care, quality improvement, secondary care

## Abstract

**Background:**

Patient experience surveys are increasingly important in the measurement of, and attempts to improve, health‐care quality. To date, little research has focused upon doctors’ attitudes to surveys which give them personalized feedback.

**Aim:**

This paper explores doctors’ perceptions of patient experience surveys in primary and secondary care settings in order to deepen understandings of how doctors view the plausibility of such surveys.

**Design, setting and participants:**

We conducted a qualitative study with doctors in two regions of England, involving in‐depth semi‐structured interviews with doctors working in primary care (*n* = 21) and secondary care (*n* = 20) settings. The doctors in both settings had recently received individualized feedback from patient experience surveys.

**Findings:**

Doctors in both settings express strong personal commitments to incorporating patient feedback in quality improvement efforts. However, they also concurrently express strong negative views about the credibility of survey findings and patients’ motivations and competence in providing feedback. Thus, individual doctors demonstrate contradictory views regarding the plausibility of patient surveys, leading to complex, varied and on balance negative engagements with patient feedback.

**Discussion:**

Doctors’ contradictory views towards patient experience surveys are likely to limit the impact of such surveys in quality improvement initiatives in primary and secondary care. We highlight the need for ‘sensegiving’ initiatives (i.e. attempts to influence perceptions by communicating particular ideas, narratives and visions) to engage with doctors regarding the plausibility of patient experience surveys.

**Conclusion:**

This study highlights the importance of engaging with doctors’ views about patient experience surveys when developing quality improvement initiatives.

## Introduction

Patient surveys have become important in recent years, in part due to policy initiatives that emphasize the utility of patient feedback for quality improvement.^1^, ^2^, ^3^ In England, patient experience is measured by surveys including the General Practice Patient Survey (GPPS) in primary care and the Inpatient Survey in secondary care.^4^ At the individual doctor level, the General Medical Council (GMC) recently introduced a revalidation programme requiring doctors to collect patient feedback as supporting information in a five‐yearly quality assurance procedure through which doctors ‘revalidate’, that is, retain their licence to practise.^5^ Surveys commonly measure key aspects of patient experience, including access, continuity and communication. If appropriately validated and administered, they capture an important dimension of health‐care quality.

Existing research highlights the importance that doctors assign to patient experience in principle and the potential for positive improvements based on patient feedback.^6^, ^7^ This work has also identified numerous challenges, including concerns about sample size and representativeness, respondent bias, reliability and validity of survey instruments, lack of clarity about the purpose of surveys, contextual sensitivity and challenges of interpreting patient feedback.^3^, ^6^, ^7^ Taken together, research shows that these and other concerns have limited the impact of patient feedback in both quality improvement and quality assurance modalities.^4^, ^7^

With some exceptions,^8^, ^9^ few researchers have focused upon doctors’ engagements with surveys administered at the individual doctor level, or upon how doctors working in different care settings engage with surveys. In this paper, we draw upon qualitative data to explore attitudes towards patient survey feedback on the part of doctors working in primary care (general practices) and secondary care (hospital outpatient clinics) settings. Rather than attempting to uncover ‘inherent’ features of patient experience surveys in general, our analysis focuses on the extent to which individual doctors regard surveys as plausible foundations for quality improvement objectives.

## Methods

### Data collection

We conducted 41 semi‐structured face‐to‐face individual interviews with doctors in primary (*N* = 21) and secondary care (*N* = 20) between December 2012 and September 2014, focusing on doctors’ attitudes to patient experience in the light of recently conducted individual doctor‐level patient experience surveys. Interviews with GPs (general practitioners) were conducted in 14 general practices across two regional areas in England. These practices were part of a larger group of 25 practices participating in a research project on patient experience, purposively selected to provide a range of practice characteristics including location, size, socio‐economic deprivation, geographical location and practice‐level survey scores generated through the national GP Patient Survey (GPPS).^8^ In these 25 practices, we conducted an individual doctor‐level postal survey of patients who had attended a face‐to‐face consultation with a GP in the previous 3 weeks.^10^ Each GP received an individual report with summary statistics and free‐text comments. While this particular survey was carried out by the study team, it is comparable in content and methodology to the surveys that doctors are required to carry out for revalidation purposes. In this wider sample of 25 practices, we aimed to interview two doctors from practices with low GPPS scores and one from each medium‐ and high‐scoring practice. The present study excluded 19 interviews conducted with GPs prior to the introduction of revalidation in December 2012, leading to 21 interviews in 14 practices. Individual GPs were identified randomly within each practice and approached one by one for consent to participate. Interviews were conducted with GPs by three individual researchers (OB, JB, CF).

Interviews with secondary care doctors were conducted in six outpatient clinics in a large regional hospital located in the same area as several of the GP practices. The participating clinics were approached because each had recently conducted an individual doctor‐level survey, using a questionnaire adapted from the national GMC patient questionnaire.^5^ Doctors received an individual report with summary statistics and free‐text comments. All doctors working within the six outpatient clinics were approached by a researcher; recruitment took place on the basis of their availability for interview. The numbers of doctors recruited in each clinic were as follows: dermatology (*N* = 2), gynaecology (*N* = 3), neurosurgery (*N* = 1), plastic surgery (*N* = 4), renal medicine (*N* = 7) and rheumatology (*N* = 3). With the exception of one doctor in training, all participating doctors were consultants. Interviews were conducted by one researcher (CF).

An interview topic guide was developed in the light of existing literature to focus on individual‐level patient experience surveys, modified slightly where necessary to incorporate emerging themes and to align with contextual features of primary and secondary care. Interviews lasted between 20 and 60 min.

### Data analysis

Interviews were digitally recorded with written consent and transcribed verbatim. NVivo software (QSR International Pty Ltd., Version 10, 2012, Cheshire, UK) was used to categorize the data, with an initial coding framework discussed among the team and revised for application to hospital interviews as well as GPs. We analysed these interviews using thematic analysis^11^ oriented towards notions of plausibility and contradiction, with particular regard to the coexistence of contradictory views.

## Findings

Our analysis found that doctors displayed contradictory views regarding the plausibility of patient surveys, leading to complex and, on balance, negative engagements with patient feedback. We outline two main identified dimensions of doctors’ views towards patient experience surveys. The first relates to doctors’ views of patients’ motivations and competence as survey respondents. The second relates to doctors’ views of surveys from a quality improvement perspective.

## Patients and surveys

Many interviewees expressed contradictory views of patients as survey respondents, combining in the same interview positive views of patients’ motivation and competence with a range of somewhat more emphatic negative views. For example, one GP emphasized patients’ capacity to identify specific problems:

I think the patient feedback is really important… You've got to actually listen to what are patients saying, [e.g.] they are telling us through this [feedback] that the system currently in place for booking appointments… is not working for them. GP2

The same GP also stressed, however, the ways in which patients’ comments were often of little use for improving care quality, especially at the individual doctor level:

When I read the comments it was just a diatribe of accusations against the practice as a whole… [I]n terms of my individual practice it gives me no feedback at all… [The] majority of the comments on the appointment system and on lack of [relational] continuity [were all]… issues that we are totally aware of.

This pattern of strong positive statements coexisting with strong negative statements was repeated in a majority of the interviews, with 17 GPs (from a total of 20) and 14 hospital doctors (from a total of 21) describing marked contradictory views with regard both to patients as survey participants and the use of patient experience surveys for quality improvement.

### Positive attitudes

In terms of positive remarks, first, both GPs and hospital doctors emphasized the centrality of the doctor–patient relationship and the utility of receiving patient feedback. For example, a GP interviewee described the doctor–patient relationship as an ‘adult‐to‐adult’ relationship in which patients know more about some things than doctors, and in which doctors need to listen to patient feedback:

the only way you're going to know whether you're doing your job properly… it's listening to what the patients are telling you [in their feedback] GP4

Against this backdrop, many GPs and hospital doctors discussed patients’ motivation and competence to provide feedback in more detail. One GP discussed how patients’ feedback showed that they were reflecting upon their experience before communicating it through free‐text comments:

They're… thinking ‘Well, actually, what *do* we think of the [practice]?’… rather than just at the time when they're desperate for an appointment and frustrated, you know, to think actually… what things at the [practice] do they actually value. GP9

As such, many doctors saw patients as being motivated to reflect upon and communicate their experiences.

Some doctors also expressed the view that patients are competent to judge their care. One hospital doctor, for example, noted that if her care were to be substandard, she would expect patients to highlight this in feedback:

I think if you have enough people, enough responses, then… if there was a systematic thing that you were doing wrong, you hopefully would pick it up [in patient feedback] HD3

### Negative attitudes

Less positively, doctors questioned patients’ motivations, firstly by viewing patients who provided negative feedback as doing so because they had specific grievances to express (‘if they've got an axe to grind’ [GP10]) and secondly by suggesting that patients participate in surveys in order to gain leverage over doctors. For example, one hospital doctor discussed how patients mention their participation in surveys

as a bargaining tool… to make you aware that there's a bit of paper at the end of the day… Some of them will actually say, ‘I'm watching you, doctor.’ HD4

Many interviewees also questioned patients’ ability to provide accurate and relevant feedback. Six principal criticisms of patients emerged from our findings; singly and/or collectively, doctors saw these as undermining patients’ ability to provide accurate feedback:

1. *Positive bias*: the tendency of patients to give strongly positive feedback regarding doctors: ‘“Sorry to take up your time” is a classic quote we hear all the time… that may well translate into giving positive feedback’ (HD4).
2. *Negative halo effects*: patients ascribing negative characteristics to consultations because of other negative experiences. As one GP described, patients may carry an ‘initial bad experience’ with the practice reception ‘all the way through… into the consulting room as well… it affects all of your feedback’ (GP6).
3. *Failure to understand surveys*: one GP noted that ‘because [patients] don't understand the questionnaire, they might tick whatever box … [i.e. on a random basis]; and that's the reason we don't get true results’ (GP19).
4. *Inconsistency*: several doctors emphasized that different individual patients could give different feedback despite having similar experiences. One hospital doctor, a surgeon, gave the example of two patients who had had an identical operation: ‘One patient I think gave us a four and one gave us a six out of six… it depends really on what their mood is, how worried they are’ (HD9).
5. *Inability to evaluate clinical competence*: doctors from both settings highlighted patients’ inability to judge their clinical competence. For example, one hospital doctor, having first stated that what ‘the patient feels or thinks… at the end of the day [is] my priority’, later added that ‘in terms of my ability to think clinically… I would go on what my consultant colleagues think [rather than patient feedback]’ (HD16).
6. *‘Good doctors, bad feedback’*: doctors felt that good care (e.g. refusing to prescribe antibiotics) could result in negative feedback if it did not meet patients’ preferences: ‘if you're giving out bad news to your patient and not telling your patient what they want to hear, then they perceive that as poor communication’ (HD3).

Overall, while individual doctors often expressed both positive and negative views of patient feedback, negative views tended to dominate (Table 1).

**Table 1:** Doctors’ attitudes to patients’ motivation and competence

Doctors’ attitudes	Positive	Negative
Category:
1. Patient motivation	Willing to take time to provide feedback	Axe‐grinding
	Used to providing feedback in other spheres	Desire to influence doctors
2. Patient competence (i.e. their ability to provide accurate and relevant feedback)	Able to recognize good quality care/improvements	Positive bias
		Negative halo effects of other clinic/survey experiences
		Inability to understand survey instruments
		Inconsistent judgements
		Lack of clinical knowledge
		Good doctor/bad feedback

## Patient experience surveys and quality improvement

### Positive attitudes

Doctors emphasized the potential for patient experience surveys to facilitate quality improvement. A number of doctors described negative feedback as having more utility for change than positive feedback. As one hospital doctor noted:

we learn more from people who complain, because we all blindly assume that we're fantastic and we're not in everything… you don't like to receive the criticism, but that's the only way… we're going to improve. HD10

Furthermore, a number of doctors in both settings discussed the potential for quality improvement to be driven by doctors’ competitiveness with regard to colleagues’ performance and/or benchmarked data (i.e. data supplied alongside comparative figures for comparable surveys undertaken in the past or elsewhere). Hospital doctors particularly emphasized their tendency to be ‘a bit competitive about it’ (HD4) and noted how benchmarked survey data could aid quality improvement by stimulating reflection by ‘start[ing] up all sorts of other conversations… saying actually we all want to be over here, how do we do that?’ (HD5).

Overall, interviewees saw the potential for survey‐based quality improvement in three main areas:

1. *Reminders of core proficiencies*, especially communication skills and basic tasks such as ensuring that patients are satisfied with the consultation before they leave. One GP said, ‘I think it flags up … the initial consultation tips that you *think* you do that perhaps you don't always’ (GP5).
2. *Reinforcements of known problems (and providing evidence to support change),* often at the clinic or practice level. For example, one hospital doctor noted how surveys provided support for change by giving doctors the evidence they needed to persuade nurses to answer patients’ buzzers in the hospital: ‘we know that nurses are so busy, [it's] difficult to go over and see the patient when they press the buzzer… everybody knows that and we try and do better, but actually having it in black and white actually helps deal with it’ (HD13).
3. *Unexpected issues documented in free‐text comments*. These were often seen as providing more useful material than numerical feedback, which was seen as overly positive. One hospital doctor described an appointment in which he had asked a new patient about the swelling in her limbs, not having realized that the patient was wearing prostheses following double amputations. The mistake was highlighted by the patient in free‐text comments in a subsequent survey, together with a request that doctors examine medical notes with more care before appointments. After noting that he now does this, he then stated that ‘the whole process [of the survey]… was useful, if nothing else, because [of] this one specific example of change to my practise’ (HD7).

### Negative attitudes

As with doctors’ views of patients as survey respondents, doctors’ positive attitudes were undermined by a plethora of sceptical views. For most of our interviewees, this led to an ambiguous but overall negative picture in which the value of surveys for quality improvement was placed in severe doubt. This is in line with preceding research in other fields that describes the scepticism of primary care staff towards patient experience surveys and the challenges of interpreting survey data.^10^, ^12^, ^14^ As well as negative views of patient motivations and competence, GPs and hospital doctors added several reasons for discounting surveys as quality improvement tools. Broadly, these concerns fell into five categories:

1. *Concerns about the validity and reliability of surveys* on the basis of factors including low response numbers, biased samples and problematic administration methods. The outpatient clinic survey was notable for the low numbers of responses (often <10 respondents), and as such, it was unsurprising that many hospital doctors remarked that they would assign greater importance to the feedback if their response rates had been higher, for example: ‘I think if more than 50 people said I was X or Y, I think I'd put more weight on it’ (HD12). However, GPs also expressed concern about response numbers despite having far higher numbers of respondents (with a mean of 71).
2. *Difficulties surrounding interpretation*, especially in hospitals owing to the lack of benchmarking data, but also more widely, regarding the separation of statistics from free‐text comments and thus the difficulty of interpreting patients’ rationale for specific responses. As one GP remarked, ‘basically if there was a problem there [in the numbers] I'd look towards addressing that, but I couldn't really find a comment which was associated with that … so I found it quite difficult’ (GP1). As research has found in other contexts, feedback presented to health‐care professionals without expert facilitation can be difficult for them to interpret and act upon.^13^
3. *Issues of context*. Doctors raised concerns about specific features of clinical encounters (e.g. different outpatient clinics) which could undermine the utility of patient feedback for quality improvement. One surgeon stated that ‘this [survey] wouldn't apply to a lot of my patients because a lot of the questions are not relevant… about treatments, drugs given, test results and medication, because most of my patients, being babies, don't have any of those’ (HD11). Hospital doctors also expressed concerns about the timing of survey administration, suggesting that administering a survey at a bad time of day or during particular weeks could lead to worse findings. In the primary care setting, some GPs who worked in deprived areas also felt that surveys did not take sufficient account of the possibility of some population groups giving systematically more negative feedback than other groups: ‘sometimes I think you have a survey and I don't think it's a true reflection of where you are, your demographics. And I think that can be a problem’ (GP11).
4. *Anxiety about negative feedback*. A number of doctors in both outpatient clinics and (especially) general practice discussed actual or potential anxiety arising as a result of negative feedback, making them less likely to adopt a positive attitude towards improving their care. Many GPs described feeling upset following negative feedback, for example: ‘I find it quite difficult, because I'll always take it quite personally’ (GP3). Several hospital doctors also alluded to the potentially upsetting nature of negative patient feedback, for example: ‘if there is a negative comment you feel, oh God, I really worked hard for this, and then all they [the patient] can say is this’ (HD9).
5. *The risk of raising patient expectations* by introducing a consumerist element more associated with customer relations than medicine. As one GP noted, ‘it's like TripAdvisor, everything, everybody's being rated’ (GP8). As several doctors noted, it is not always possible to meet these rising expectations, especially with regard to resource‐related issues such as out‐of‐hours appointments or (in the hospital setting) scans and tests; consequently, surveys may encourage patients to expect changes that are impossible to implement in practice, leading in turn to negative patient feedback. Thus, if quality improvement is evaluated at least in part on the basis of patient experience surveys, surveys themselves may render evidence of improvement less likely.

Overall, negative views of the potential contribution of patient surveys to quality improvement agendas dominated our findings (Table 2).

**Table 2:** Doctors’ attitudes to patient experience surveys as quality improvement tools

Positive	Negative
Value of reflecting upon patient feedback	Discounting of patient motivations and competence
Value of competition between doctors on the basis of survey feedback	Concerns about the validity and reliability of surveys
Reminders of core proficiencies	Difficulties surrounding interpretation
Reinforcements of known problems (and providing evidence to support change)	Issues of context
	Anxiety about negative feedback.
Unexpected issues documented in free‐text comments	Risk of raising patient expectations

## Discussion

Our study explored doctors’ engagements with patient experience surveys in primary and secondary care settings. We discussed doctors’ views about surveys with regard to, first, patients considered as survey respondents and, second, the potential of patient feedback to facilitate quality improvement. While doctors endorsed patients’ motivations for participating in surveys and their competence to provide relevant feedback, these notions were outweighed by doctors’ emphasis upon what they saw as patients’ questionable motivations and lack of competence. Consequently, doctors appear to view patients in a contradictory fashion – that is as being simultaneously competent and incompetent at evaluating doctors and as being both accurate reporters of experience and inevitably biased commentators. Likewise, while doctors emphasized the potential utility of patient feedback for quality improvement, they also presented numerous factors which undermined this agenda. Overall, doctors’ engagements with patient experience surveys were highly contested, problematic and inconsistent, with the majority of interviewees appearing to consider more than one interpretation of patient experience surveys as plausible at the same time. Nevertheless, doctors did not see all interpretations as equally plausible. As discussed above, they tended to settle on negative views of patients (considered as survey respondents) and of patient experience surveys, thus undermining the potential for reflective change and quality improvement in response to patient feedback (in line with previous research).^6^, ^7^, ^8^

### Quality improvement or quality assurance?

Doctors’ scepticism towards patient surveys as quality improvement tools raises the question of whether surveys might better be understood as tools for quality assurance purposes.^8^ However, previous research shows that doctors are also sceptical about the use of patient experience surveys for quality assurance, regarding them as incapable of identifying malicious doctors and/or as potentially facilitating political meddling with health‐care services.^7^ Consequently, it seems that doctors’ engagements with patient experience surveys are characterized by contradiction, whether the overarching agenda is quality improvement, quality assurance or both (as in the GMC's current revalidation programme). From this perspective, rather than viewing surveys as ideally or inherently suited for quality assurance or quality improvement, it is perhaps more productive to view them as inherently contested enterprises capable of application within a range of wider agendas, and whose success or otherwise (in terms of the stated objectives of those agendas) depends heavily on contextual characteristics of doctors’ engagements with survey findings as they are disseminated and embedded in local settings. This notion focuses attention on how future interventions might engage with doctors’ experiences of survey findings in a more productive manner in specific settings.

### Engaging with doctors through sensegiving dialogue

While our findings show the problematic nature of doctors’ engagements with patient experience surveys, they also suggest the possibility of productive change in the future by building on some of the positive views that doctors already hold regarding patients and surveys. We link this potential to notions of ‘sensegiving’, or attempts to influence perceptions by the communication of particular ideas, narratives and visions.^14^ To engage in sensegiving is to create the possibility of change in a target audience by suggesting that existing interpretive schemes (e.g. current notions of plausibility) may no longer be useful. Once this takes place, an opportunity exists to ‘articulate and advocate [a new] vision or preferred interpretative scheme’.^14^ Importantly, sensegiving processes can be understood as meaningful interactions between stakeholders rather than a top‐down process of ‘educating’ key players through the provision of additional information. Thus, Clark and Geppert define sensegiving in terms of actors ‘respond[ing] meaningfully to and thereby influenc[ing] the behavior of others’.^15^

In the patient survey context, opportunities exist for policymakers, managers and lead clinicians to respond meaningfully to doctors’ perceptions of patient feedback surveys (as discussed above), on the basis that such perceptions are equally as important as inherent properties of surveys (e.g. their reliability, validity and other psychometric characteristics). For hospital doctors and GPs to see quality improvement on the basis of patient feedback as plausible, our findings suggest they would need to be persuaded simultaneously of: patients’ evaluative competence and disinterestedness; the possibility of interpreting feedback meaningfully (e.g. through the provision of benchmarked data); the ability of survey instruments to take account of contextual factors (e.g. to allow for differences between distinct care settings); adequate provision of support for doctors receiving negative feedback; and assurance of measures to limit the risk of raising patient expectations.

**Table 3:** Plausibility of patient experience surveys: limiting factors and potential foci for sensegiving dialogue

Factors inhibiting plausibility of interpretations favouring quality improvement		Foci for potential sensegiving dialogue
1. Views of patients	Not disinterested evaluators	Nature of doctors’ personal engagement with patients; psychometric bases of validity/reliability
	Incompetent evaluators	Nature of doctors’ personal engagement with patients; survey administration process; instructions given to patients on survey instruments
2. Views of surveys	Difficulties of interpreting feedback	Facilitated feedback for individual doctors/groups of doctors, embedded within wider local change programmes; additional information on feedback material (e.g. benchmarking data)
	Lack of contextual sensitivity	Potential for development/validation of tailored survey instruments for different care settings
	Anxiety regarding negative feedback	Nature of support provided to individual doctors concerned about negative feedback
	Risk of raising patient expectations	Potential to limit frequency of survey administration to minimum necessary, except where raising patient expectations is intended

In each of these arenas, as presented in Table 3, potential exists for sensegiving dialogue to take place with doctors in order to highlight and discuss factors currently inhibiting the plausibility of patient experience surveys for quality improvement purposes. Such dialogue could take place in a number of different formats, including online discussions, dedicated sessions during initial training and at continued professional development (CPD) events, or through local networks of clinicians and others in (e.g.) general practice and hospitals. By facilitating and engaging in such dialogue, policymakers, managers and other relevant stakeholders can attempt to engage positively and meaningfully with doctors’ current views about patient surveys. The aim of sensegiving processes would not be to encourage doctors to adopt an attitude of uncritical acceptance towards patient experience surveys, nor that doctors should only hold views that are wholly positive or negative (or consistent with those of other doctors), but rather to explore in depth some of the key barriers that surveys face in terms of plausibility for quality improvement.

## Conclusion

This paper has explored the ambiguities in doctors’ attitudes to patient experience surveys across primary and secondary care, focusing on doctors’ views regarding the plausibility of survey findings. While policy developments over the past decade have increasingly emphasized the importance of patient experience surveys for quality improvement, our findings suggest that this agenda faces significant challenges in terms of doctors’ inconsistent and highly critical engagements with patient feedback. Doctors criticize patients’ motivations and competence at the same time as emphasizing patient‐centred care, and undermine the potential for survey‐based quality improvement while also highlighting the importance of patient feedback. Doctors working in two different health‐care settings demonstrate similarly complex and contradictory attitudes towards the plausibility of patient feedback – attitudes that are likely to constrain the potential impact of patient experience surveys on care delivery. In response, we highlight the need for ‘sensegiving’ dialogue on the part of policymakers, managers and clinicians in order to engage with doctors’ concerns about the plausibility of surveys.
